# Biomechanical Effects of Horizontal, Vertical, and Combined Misfits in Full-Arch Implant-Supported Titanium Frameworks: A Three-Dimensional Finite Element Analysis

**DOI:** 10.3390/jfb17090478

**Published:** 2026-09-19

**Authors:** Hale Arikan Kalayci, Mustafa Baris Guncu

**Affiliations:** 1 Department of Prosthodontics, Faculty of Dentistry, Başkent University, Ankara 06790, Turkey; 2 Department of Prosthodontics, Faculty of Dentistry, Hacettepe University, Ankara 06100, Turkey; barisguncu@hacettepe.edu.tr

**Keywords:** finite element analysis, implant-supported prosthesis, titanium framework, framework misfit, biomechanical stress, peri-implant bone

## Abstract

This study evaluated the effects of misfit type, magnitude, and location on the stress distribution in full-arch screw-retained implant frameworks. A maxillary finite element model with four implants was analyzed under 16 scenarios, characterized by horizontal misfits of 10, 50, 100, and 200 µm; vertical misfits of 10 and 100 µm; and combined 10–10 and 100–100 µm misfits, each positioned anteriorly or posteriorly. Forced seating was simulated using prescribed displacement; no occlusal load or screw preload was applied. Von Mises stresses were evaluated in the framework, occlusal screws, and implants, and principal stresses were assessed in peri-implant bone. Framework stress increased with horizontal misfit magnitude. When the same numerical misfit value (10 or 100 µm) was applied at the same location under otherwise identical model conditions, vertical misfit produced higher framework stress than horizontal misfit, indicating a direction-dependent response associated with different seating deformation modes. The posterior 100–100 µm combined misfit produced the maximum framework (356 MPa), occlusal screw (193 MPa), and implant (250 MPa) stresses. In contrast, the maximum principal stress peaked at the posterior bone site with the anterior 200 µm horizontal misfit (V0 H200 A; 77 MPa), while the most negative minimum principal stress occurred at the posterior bone site with the posterior 200 µm horizontal misfit (V0 H200 P; −52 MPa). Among the tested scenarios, combined misfits yielded the highest framework and screw stresses, whereas implant and bone responses depended on the magnitude and location of the misfit. These findings indicate that the types, magnitudes, and locations of misfits should be considered during framework-fit assessments, with particular attention to combined misfits, before definitive seating.

## 1. Introduction

Implant-supported fixed complete dentures show high survival in edentulous patients and, when supported by four implants, may reduce the need for extensive surgery [1,2,3]. Passive fit—the absence of strain in implant components and the framework after screw preload—is considered important for limiting stress, whereas misfit is a dimensional discrepancy at the prosthetic interface [4,5,6,7]. Misfit may contribute to screw loosening or fracture, framework fracture, veneering-material chipping, and increased peri-implant biomechanical demand [4,5,6,7]; it should therefore be minimized before definitive seating.

Although implant outcomes are multifactorial and factors beyond prosthetic fit influence peri-implantitis surgery and late failure [8], misfit is a modifiable factor. Fit can be assessed by visual or tactile inspection, radiography, the one-screw test, microscopy, strain-gauge measurements, and finite element analysis (FEA) [6,9].

Framework discrepancies may be vertical, horizontal, or combined. Research has emphasized vertical gaps because they are more readily quantified microscopically, leaving horizontal effects less understood [10]. Horizontal distortion may also escape conventional vertical-gap assessment [10,11]. The often-cited acceptable range of 10–150 µm lacks strong support [12], and a recent systematic review found insufficient evidence for a universal threshold [5]. That review reported direction- and outcome-dependent ranges: vertical values of 30–160 µm and horizontal values up to 150 µm without mechanical complications, with broader ranges for biological outcomes [5]. These observations guided our directional comparison but were not treated as validated thresholds. Although absolute passivity may be unattainable, even small discrepancies can concentrate stress [13,14,15].

Clinically, considering misfit direction and magnitude may support decisions to accept, adjust, or remake a framework, whereas reliance on a visible vertical gap may overlook horizontal or combined discrepancies. Combined misfit is especially relevant because simultaneous vertical and horizontal components may produce deformation and stress transfer that cannot be predicted from either component alone.

FEA permits controlled isolation of geometric and mechanical variables and component-level stress estimation that is difficult to obtain directly. Rutkunas et al. evaluated vertical misfits of 50–150 µm and horizontal misfits of 35–100 µm in two-implant zirconia frameworks in vitro, whereas full-arch FEAs by Bhering et al. and Dayan and Geckili evaluated implant configuration or framework material without predefined misfit [16,17,18]. Misfit-specific FEAs modeled horizontal displacements of 10–200 µm in a two-implant overdenture bar and terminal gaps of 0–150 µm in a three-implant, five-unit prosthesis [19,20]; a recent two-implant pilot examined a single translational misfit [21]. Thus, evidence for fixed full-arch frameworks remains limited and, to our knowledge, multiple magnitudes of horizontal, vertical, and combined misfit, including lower-range combined discrepancies, have not been systematically compared at anterior and posterior sites within one model. This study therefore quantified the effects of misfit type, magnitude, and location on stresses in a full-arch titanium framework–occlusal screw–implant–bone complex. The null hypothesis was that these factors would not affect von Mises stresses in the framework, occlusal screws, and implants or maximum and minimum principal stresses in peri-implant bone.

## 2. Materials and Methods

Bone models were reconstructed from Visible Human Project computed tomography (CT) data in Digital Imaging and Communications in Medicine (DICOM) format (U.S. National Library of Medicine, Bethesda, MD, USA); the modeling report specified a reconstruction resolution of 0.33 mm. The DICOM datasets were imported into 3D Slicer (version 5.6; Surgical Planning Laboratory, Brigham and Women’s Hospital, Boston, MA, USA; open-source software) and segmented using appropriate Hounsfield unit thresholds to generate three-dimensional (3D) surface models, which were then exported as standard tessellation language (STL) files. The resulting maxillary models were processed in Blender (version 4.5 LTS; Blender Foundation, Amsterdam, the Netherlands); in particular, all models were aligned and positioned within a common coordinate system in Blender. To reflect the structurally distinct cortical shell and trabecular core of the maxilla, the two bone compartments were modeled separately [22]. A uniform 2 mm inward offset was applied to define the cortical shell, and its internal surface served as the reference for generating the trabecular core. The 2 mm thickness was not selected to reflect patient-specific anatomy but, instead, was adopted as a standardized modeling approximation, as commonly used in dental FEA-based research [23]. Anatomical measurements in edentulous maxillae vary by site and surface (approximately 1.04–2.06 mm), placing 2 mm near the upper end of the reported range [24]; furthermore, cone-beam computed tomography (CBCT) data demonstrate substantial regional variation and thinner mean crestal cortices in the maxilla [22].

**Figure 1:**
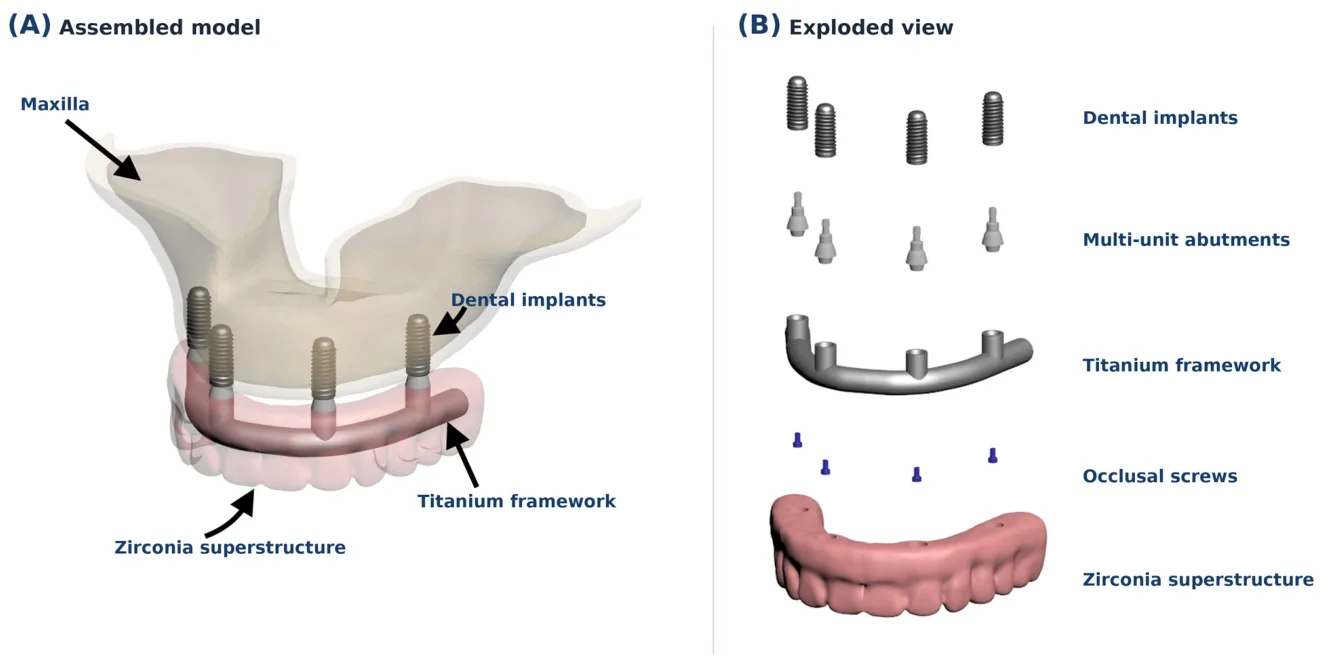
Three-dimensional finite element model: (**A**) assembled maxillary model; (**B**) exploded view of the implants, multi-unit abutments, titanium framework, occlusal screws, and zirconia superstructure.

Four implants (Straumann Bone Level, Ø4.1 mm × 10 mm; Institut Straumann AG, Basel, Switzerland) were bilaterally positioned at the maxillary lateral incisor and second premolar sites. All implants were placed axially and parallel to one another; no distal tilt was used. This standardized orientation was maintained across all scenarios in order to isolate the effects of misfit type, magnitude, and location. The implant, multi-unit abutment, occlusal screw, and titanium framework were digitally modeled in Blender, and a 12-unit zirconia fixed dental prosthesis was designed based on the anatomical reference data from Wheeler’s Dental Anatomy Atlas. The assembled model and its components are shown in Figure 1. Scenario-specific framework geometries were generated by introducing predefined discrepancies at selected framework–abutment connections. Horizontal misfit is represented by an internal-wall offset, whereas vertical misfit is represented by a marginal gap; the forced seating procedure is described below. Within the finite element model, all materials were assumed to be homogeneous, isotropic, and linearly elastic. The elastic modulus and Poisson’s ratio were respectively assigned as follows: cortical bone, 13,700 MPa and 0.30; trabecular bone, 1370 MPa and 0.30; and the 12-unit zirconia prosthesis, 200,000 MPa and 0.31 [23]. As a modeling idealization, the implant, multi-unit abutment, occlusal screw, and framework were assigned the same material properties based on commercially pure Grade 4 titanium (elastic modulus, 110,000 MPa; Poisson’s ratio, 0.35) [23,25].

Eight misfit configurations were defined according to their vertical misfit (VM) and horizontal misfit (HM) components: V0 H10, 0 µm VM and 10 µm HM; V10 H0, 10 µm VM and 0 µm HM; V10 H10, 10 µm VM and 10 µm HM; V0 H50, 0 µm VM and 50 µm HM; V0 H100, 0 µm VM and 100 µm HM; V0 H200, 0 µm VM and 200 µm HM; V100 H0, 100 µm VM and 0 µm HM; and V100 H100, 100 µm VM and 100 µm HM. To compare the effects of misfit type, magnitude, and location within the same model, each configuration was evaluated at two anatomical locations, resulting in 16 location-specific scenarios (8 × 2). In subgroup A, misfits were applied at the framework–abutment connections corresponding to bilateral lateral incisor implant sites; in subgroup P, they were applied at connections corresponding to bilateral second premolar implant sites (Table 1).

The selected misfit magnitudes were designed for parametric comparison rather than to represent clinical acceptability thresholds. Regarding the vertical magnitudes, the 10 µm condition represents the lower bound of the commonly cited but unvalidated 10–150 µm range [12], while 100 µm was selected as a representative mid-range value within the reported vertical range. The horizontal series (10, 50, 100, and 200 µm) followed the displacement levels evaluated by Spazzin et al. [19]. The 10 and 100 µm levels were included in both isolated vertical and horizontal conditions to enable matched directional comparisons. In these comparisons, all other model settings remained unchanged. Therefore, the effects of discrepancy orientation and the corresponding seating displacement direction were evaluated, rather than assuming mechanical equivalence between a vertical gap and a horizontal offset. Under the 10–10 and 100–100 µm conditions, the combined application of both components was evaluated. The 200 µm horizontal condition was retained as an exploratory upper-bound scenario; however, a 200 µm VM + 200 µm HM condition fell outside the intended range of the forced-seating analysis and, thus, was not included.

The geometric models were discretized into finite elements to generate the computational meshes. After modeling in Blender, the meshes were prepared for analysis in Altair HyperMesh (version 2024; Altair Engineering, Inc., Troy, MI, USA). Based on the mesh convergence assessment described below, triangular surface elements ranging from 0.10 to 0.25 mm were used, with the finest elements assigned to regions of expected stress concentration. Once all the model surfaces were meshed with these elements, solid meshes of the objects were created using tetrahedral elements. Mesh refinement was concentrated at the framework–abutment interface, implant components, and peri-implant cortical bone, where stress concentrations were expected to occur. Mesh quality was evaluated for all models. Elements exceeding the predefined skewness criterion (>80°) or failing to satisfy the predefined minimum-edge-length criterion were identified during the mesh quality review and corrected before inclusion in the analysis. The HyperMesh models were exported to Altair OptiStruct (version 2024; Altair Engineering, Inc., Troy, MI, USA), a Nastran-based finite element solver. The analyses were performed on an HP workstation (HP Inc., Palo Alto, CA, USA) equipped with an Intel Xeon E-2286 processor (2.40 GHz) and 64 GB of ECC RAM. The documented scenario meshes contained approximately 339,000 nodes and 1.36 million tetrahedral elements (Table 2).

A mesh convergence assessment was performed for the V100 H100 A scenario (100 µm VM + 100 µm HM) using nominal element sizes of 0.4, 0.3, 0.2, and 0.1 mm under otherwise identical boundary, geometric discrepancy, and closure displacement conditions. The peak von Mises stress in the posterior implant was monitored. Successive relative change was calculated as the absolute difference between the updated and preceding values divided by the updated value and multiplied by 100; a value below 3% was defined as indicating convergence.

Adapting the displacement-controlled framework settling approach described by Spazzin et al. [19], forced seating was implemented in four steps. First, separate unseated geometries were generated for the A and P conditions. In the A models, the prescribed discrepancy was introduced symmetrically at both anterior framework–multi-unit abutment connections while the posterior connections remained seated; in the P models, it was introduced symmetrically at both posterior connections while the anterior connections remained seated. Horizontal misfit was created as an offset of 10, 50, 100, or 200 µm between the inner wall of the framework connection and the corresponding external abutment surface. Vertical misfit was created as a 10 or 100 µm gap between the opposing framework and abutment seating surfaces; the combined conditions incorporated both components. Second, the implants and abutments remained in their original positions, and only the framework geometry was altered to establish the unseated configuration. Third, the prescribed displacement was applied directly to nodes on the upper framework surface at both discrepant connections. The horizontal component was equal to the modeled offset and was directed mesio-buccally within the horizontal plane, whereas the vertical component was equal to the modeled gap and was directed along the vertical seating axis toward the intended seated position. Both components were applied concurrently in the combined misfit configurations. Finally, each configuration was analyzed separately in OptiStruct under linear static conditions. No contact constraint was defined at the intentionally discrepant interfaces; thus, the prescribed displacement represents idealized closure of the initial mismatch. No external occlusal load or screw preload was applied.

Nodes on the superior boundary of the bone model were fixed in all three translational directions (Ux = Uy = Uz = 0), thereby preventing rigid-body motion. A symmetric boundary condition was also applied to the nodes of all model components located on the Y–Z symmetry plane: displacement normal to the plane was constrained (Ux = 0), whereas in-plane displacements (Uy and Uz) remained unrestricted. In the global coordinate system, +X and −X represent the buccal and palatal directions, +Y and −Y the posterior and anterior directions, and +Z and −Z the gingival and occlusal directions, respectively. Sixteen linear static analyses were performed using the same boundary conditions and the scenario-specific geometric discrepancy and closure displacement conditions. All other initially contacting component interfaces were assigned FREEZE contact, corresponding to bonded conditions that prevent separation and sliding at those interfaces. Together with the prescribed displacement at the misfit site, this idealization provided a common closure endpoint across scenarios. The displacement-controlled approach was selected to isolate the effects of misfit type, magnitude, and location within a standardized seating protocol, while all other model conditions were held constant. A torque-driven tightening simulation would require additional, history-dependent inputs and assumptions, including frictional contact, torque-to-preload conversion, and tightening sequence, which are beyond the scope of this study. Accordingly, the prescribed displacement represents an idealized final closure configuration rather than screw-generated preload. Each location-specific scenario was solved once. As the analyses were deterministic and did not involve repeated observations, no inferential statistical analysis was performed; as such, the results are compared descriptively.

## 3. Results

Peak posterior-implant von Mises stress increased from 120 MPa at 0.4 mm to 146 MPa at 0.1 mm. The relative change between the 0.2 and 0.1 mm meshes was 2.5%, meeting the predefined <3% convergence criterion; accordingly, a minimum element size of 0.1 mm was retained.

**Figure 2:**
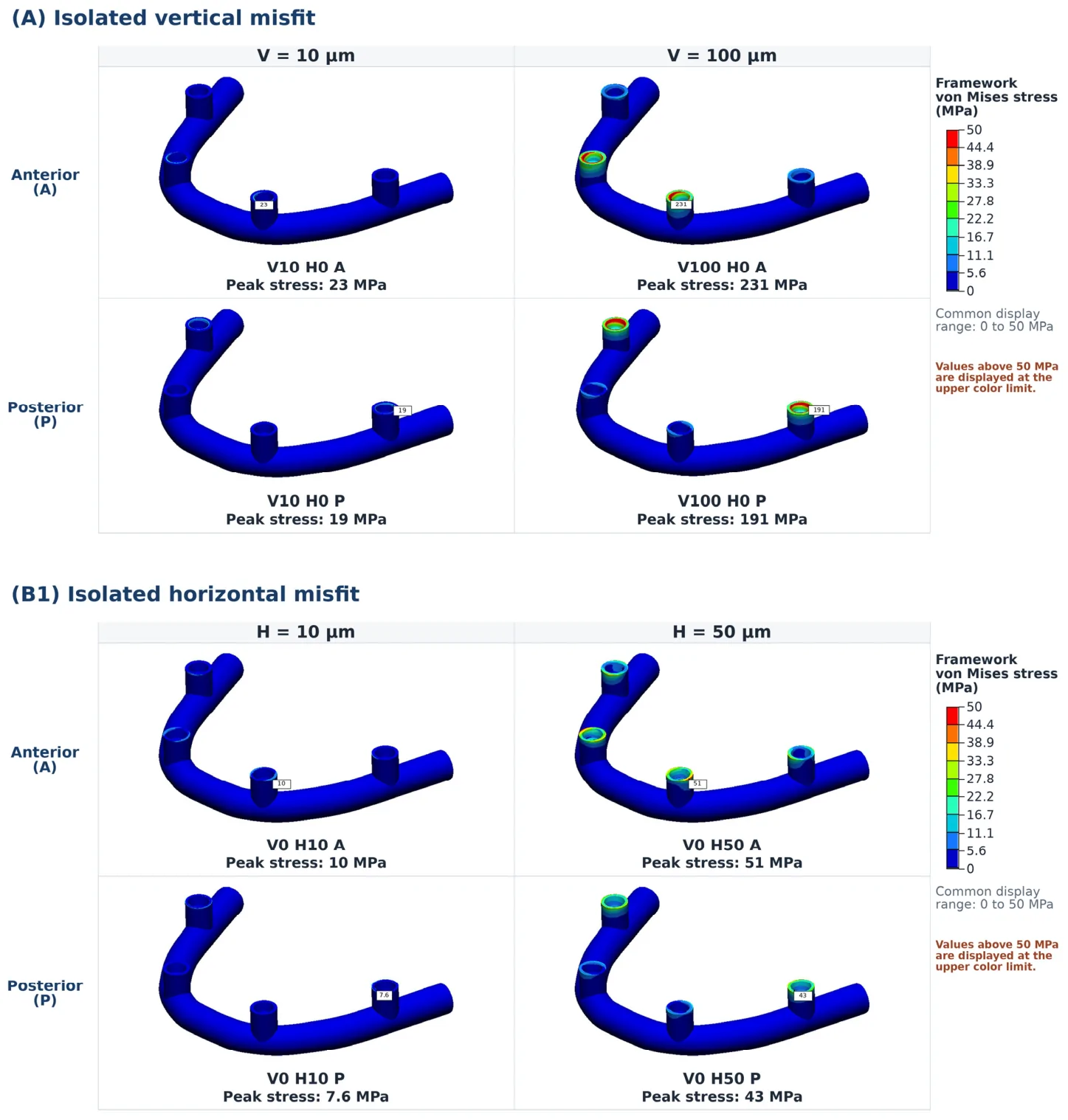

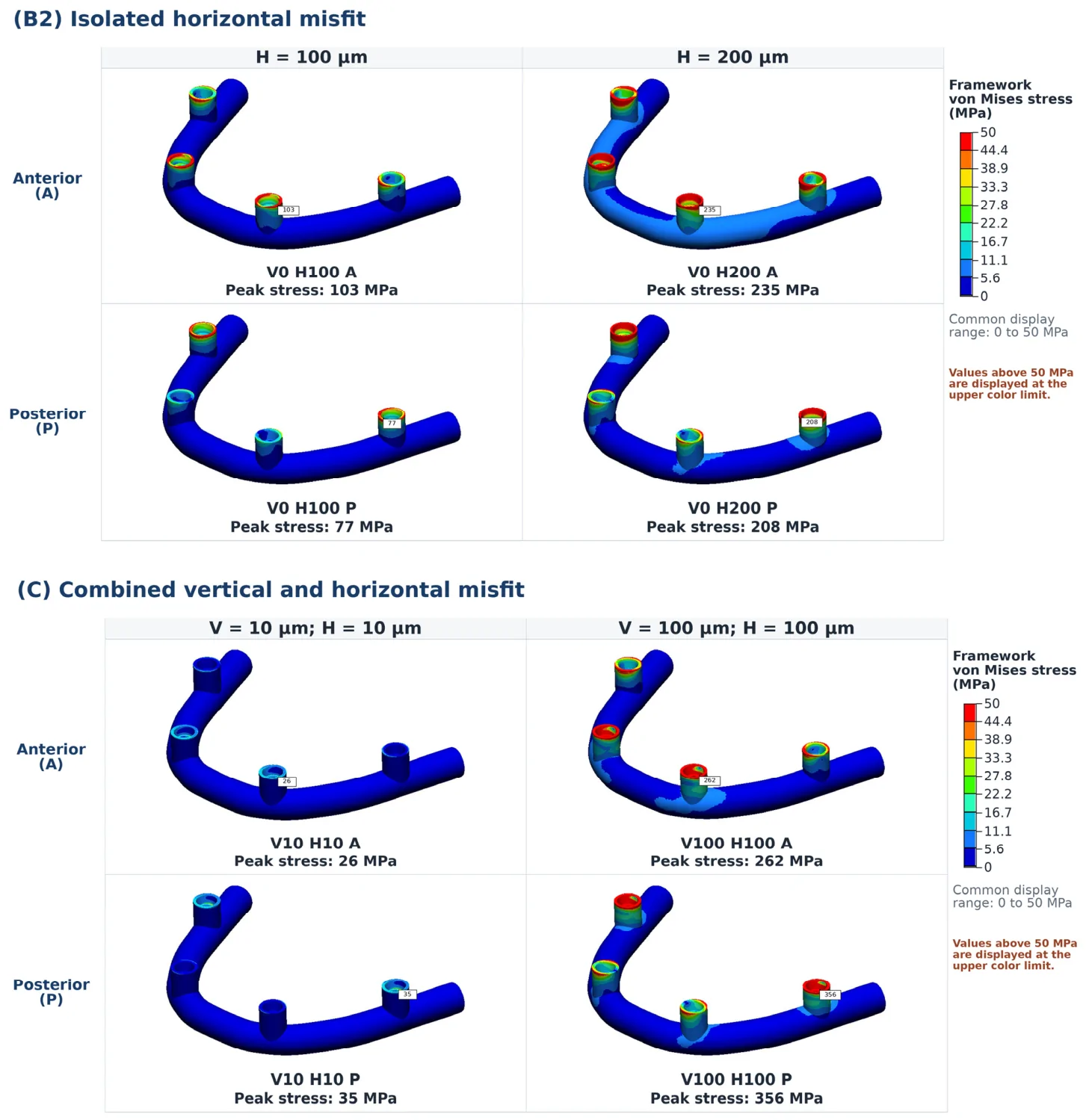
Von Mises stress distributions in the titanium framework for (**A**) isolated vertical, (**B1**,**B2**) isolated horizontal, and (**C**) combined misfit scenarios. Stress values are expressed in MPa. Boxed numbers indicate the site-specific stress values.

Framework stress increased progressively with isolated horizontal misfit magnitude at both locations. At the shared numerical values of 10 and 100 µm, vertical misfit produced higher framework stress than horizontal misfit when compared at the same location under otherwise identical model conditions. At 100 µm, the corresponding values were 231 versus 103 MPa for anterior misfit and 191 versus 77 MPa for posterior misfit. Combined misfit produced higher stresses than either isolated component at both magnitudes. Anterior misfit generated higher framework stresses in the isolated conditions, whereas posterior misfit generated higher stresses in the combined conditions. The overall maximum occurred in V100 H100 P (356 MPa) (Figure 2).

**Figure 3:**
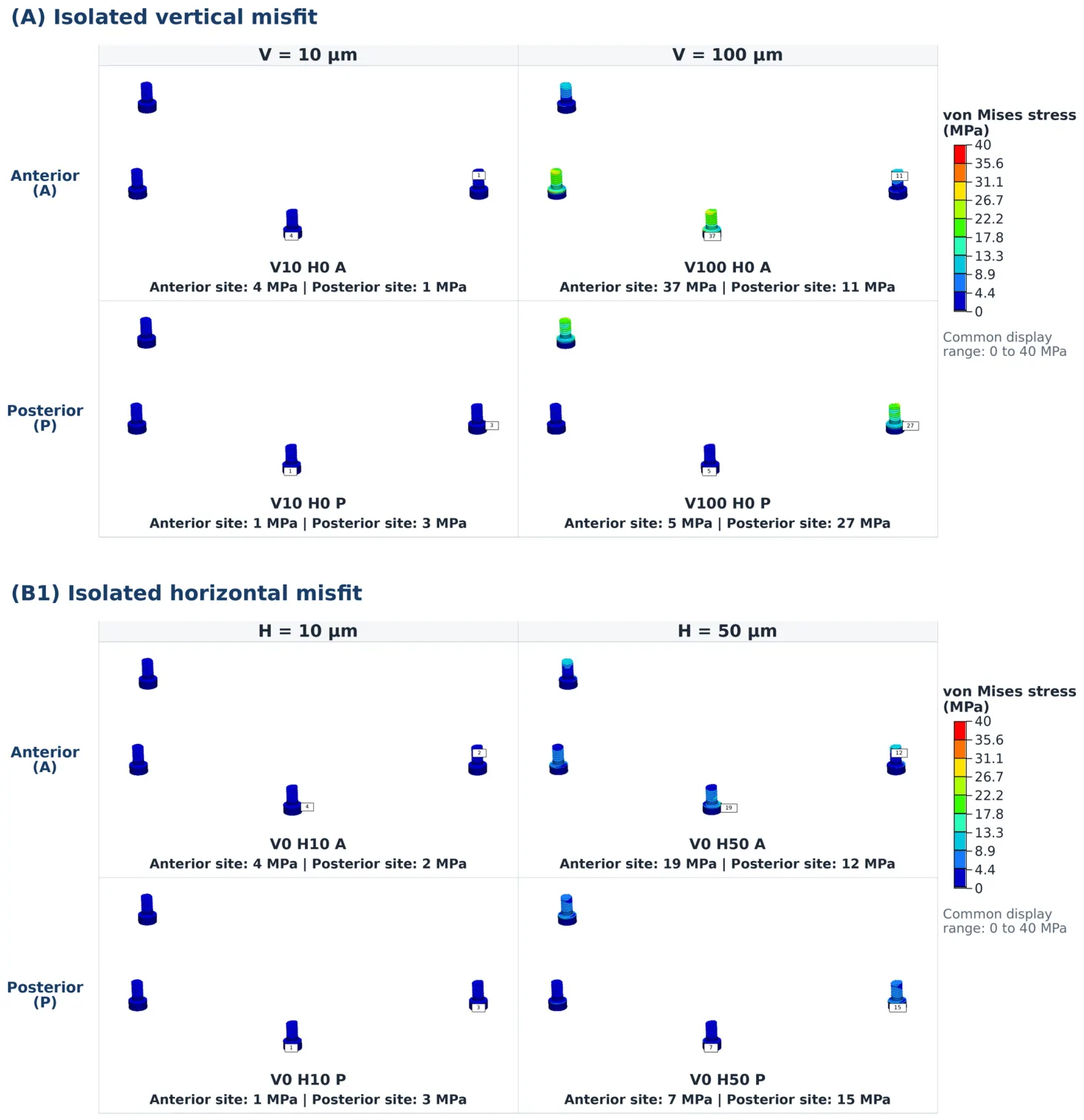

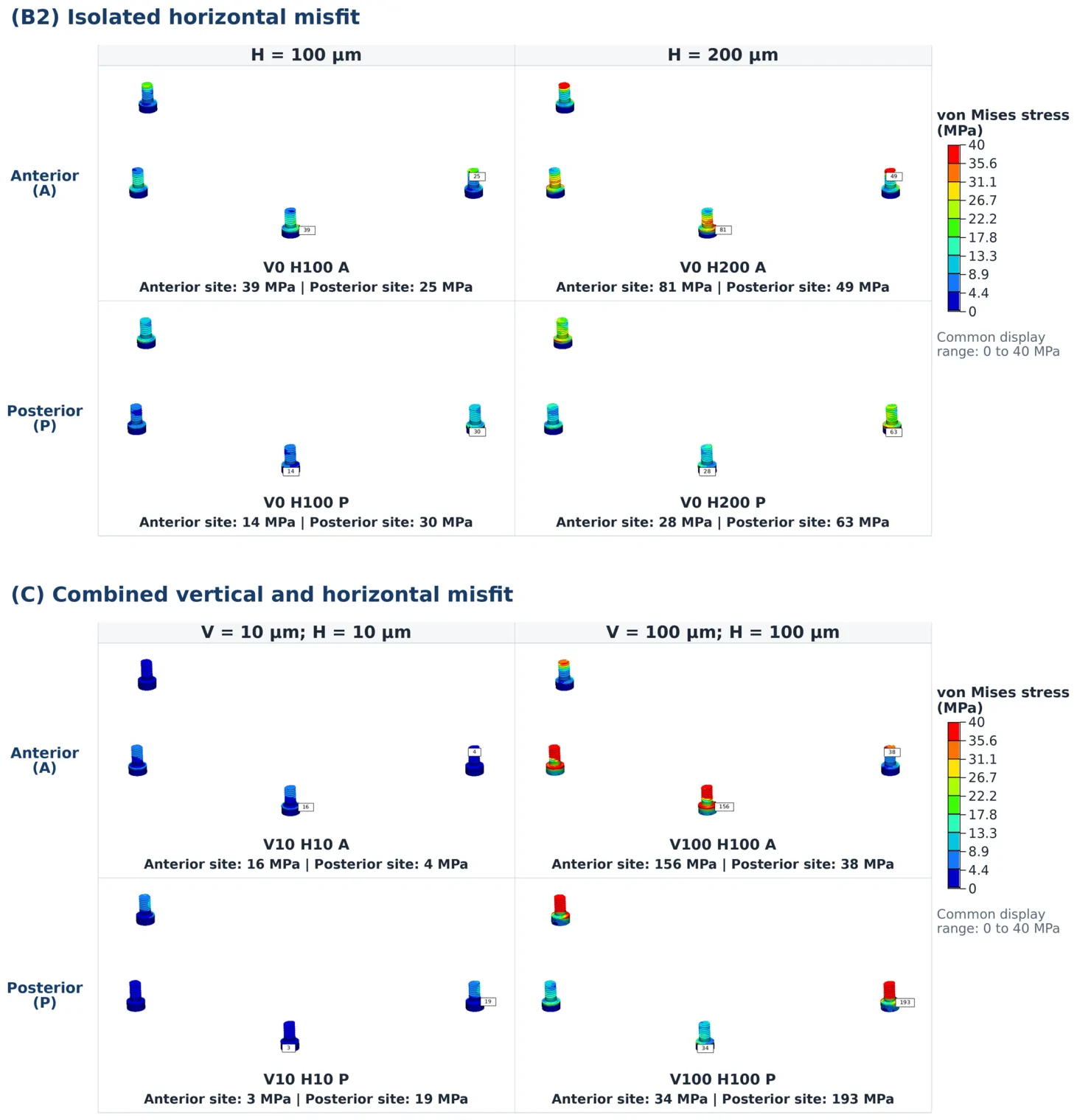
Von Mises stress distributions in the occlusal screws for (**A**) isolated vertical, (**B1**,**B2**) isolated horizontal, and (**C**) combined misfit scenarios. Stress values are expressed in MPa. Boxed numbers indicate the site-specific stress values.

Occlusal-screw stress was consistently higher at the screw corresponding to the misfit location and increased with isolated horizontal misfit magnitude. Combined misfit produced the greatest values, with the overall maximum at the posterior screw in V100 H100 P (193 MPa) (Figure 3).

**Figure 4:**
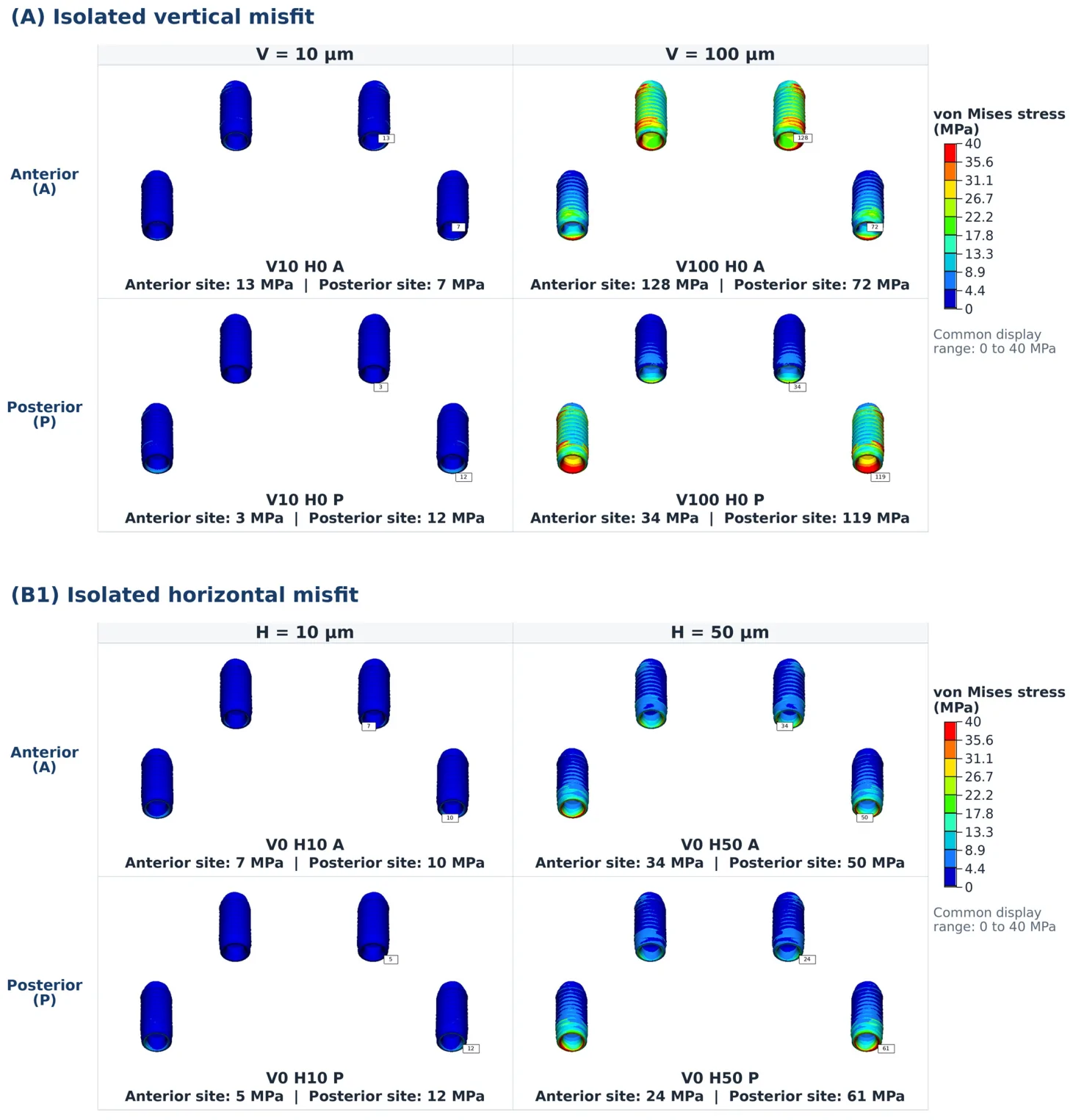

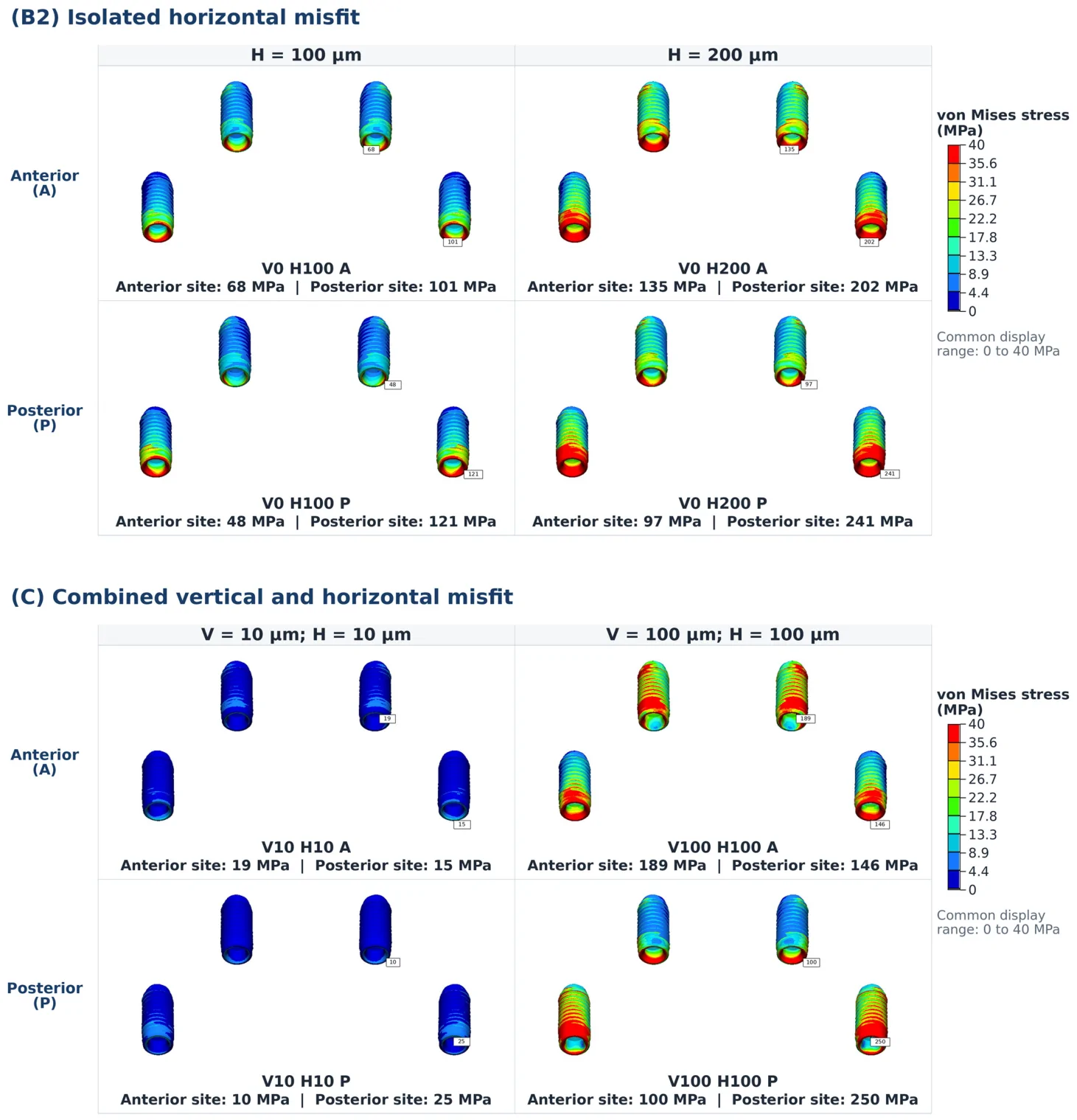
Von Mises stress distributions in the implants for (**A**) isolated vertical, (**B1**,**B2**) isolated horizontal, and (**C**) combined misfit scenarios. Stress values are expressed in MPa. Boxed numbers indicate the site-specific stress values.

Implant stress increased progressively with isolated horizontal misfit magnitude at both implant sites. The greatest values occurred in the high-magnitude combined and isolated-horizontal conditions, with the overall maximum in V100 H100 P (250 MPa), followed by V0 H200 P (241 MPa) (Figure 4).

**Figure 5:**
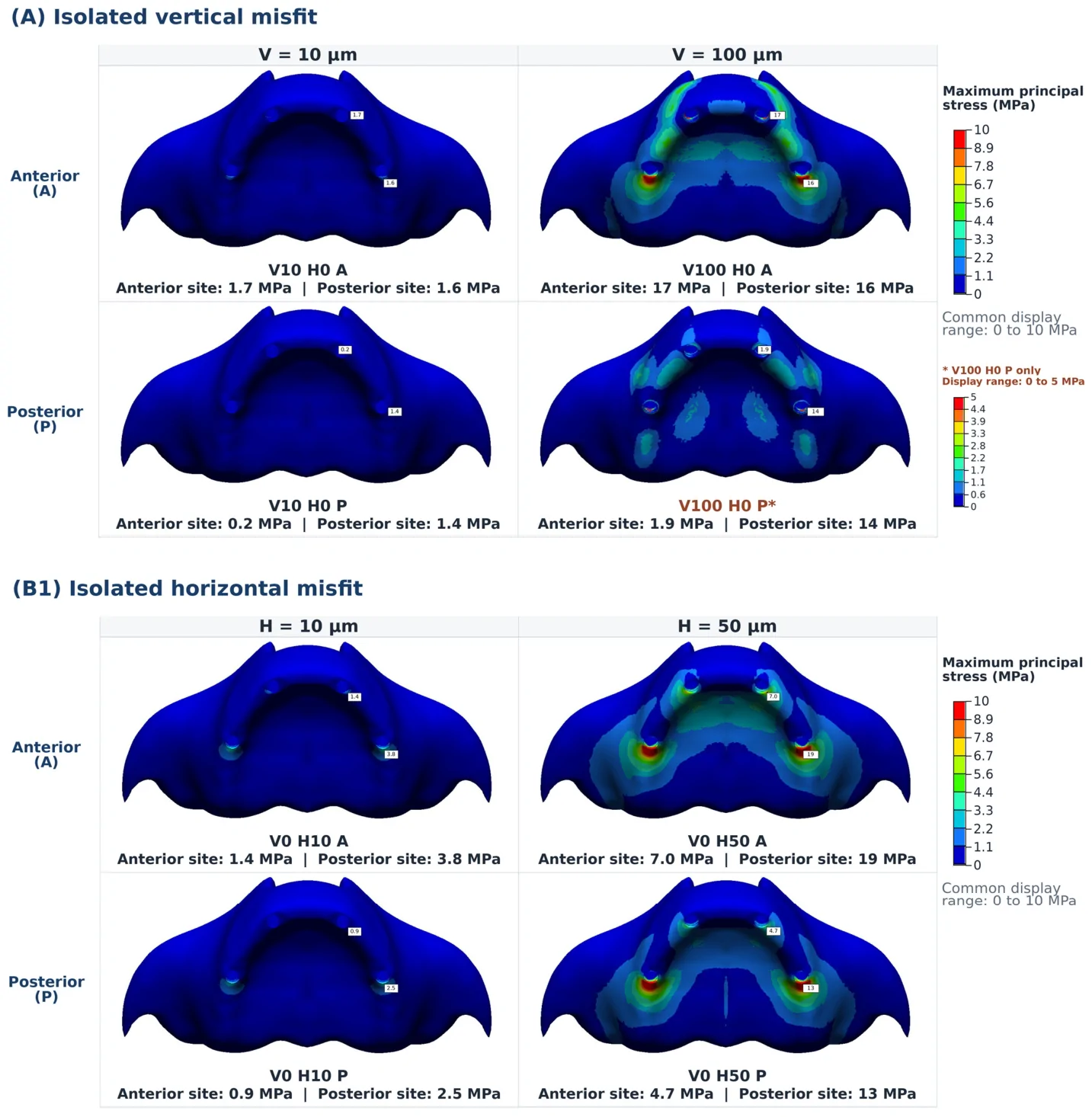

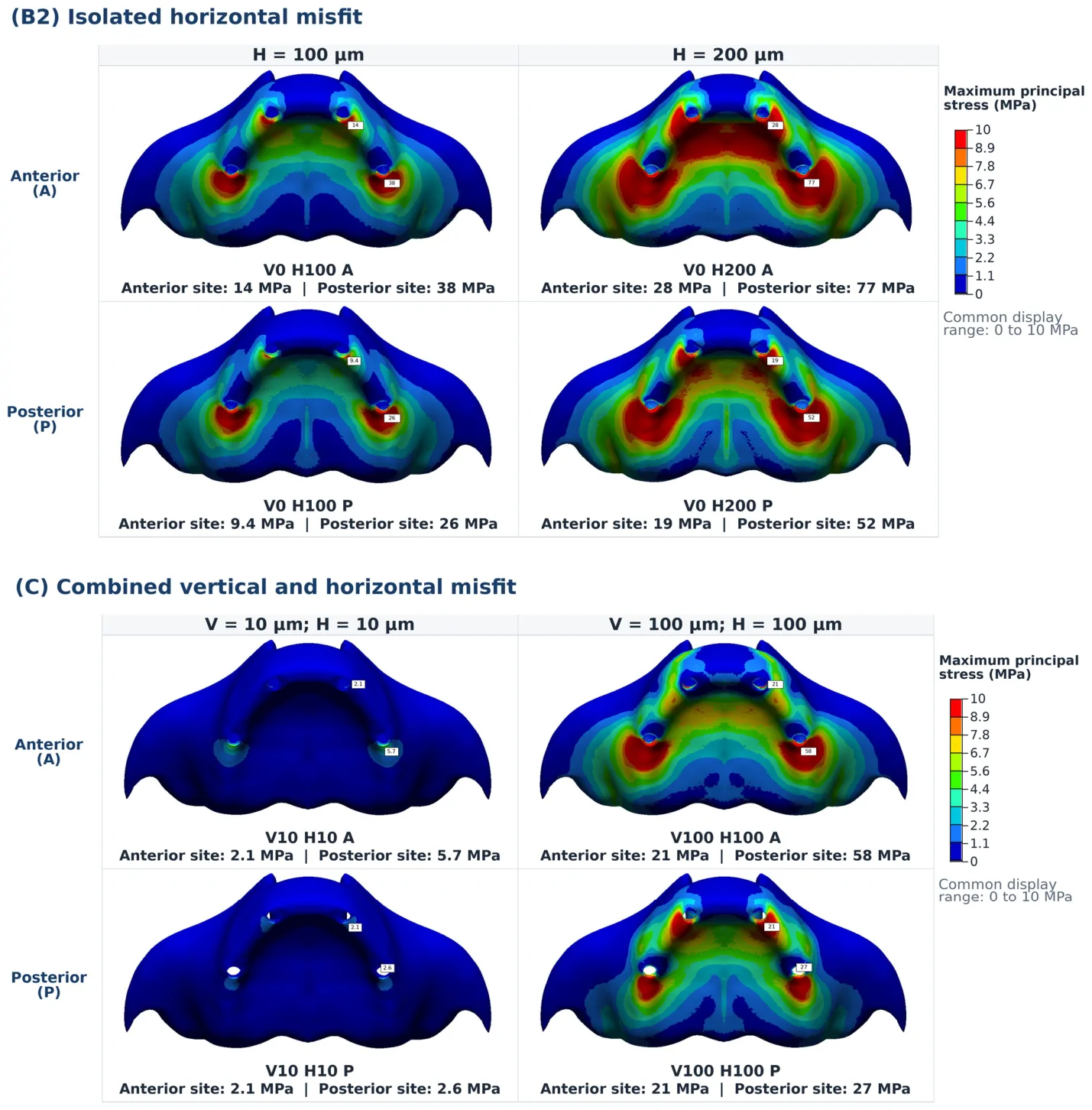
Maximum principal stress distributions in the peri-implant bone for (**A**) isolated vertical, (**B1**,**B2**) isolated horizontal, and (**C**) combined misfit scenarios. Stress values are expressed in MPa. Boxed numbers indicate the site-specific stress values.

**Figure 6:**
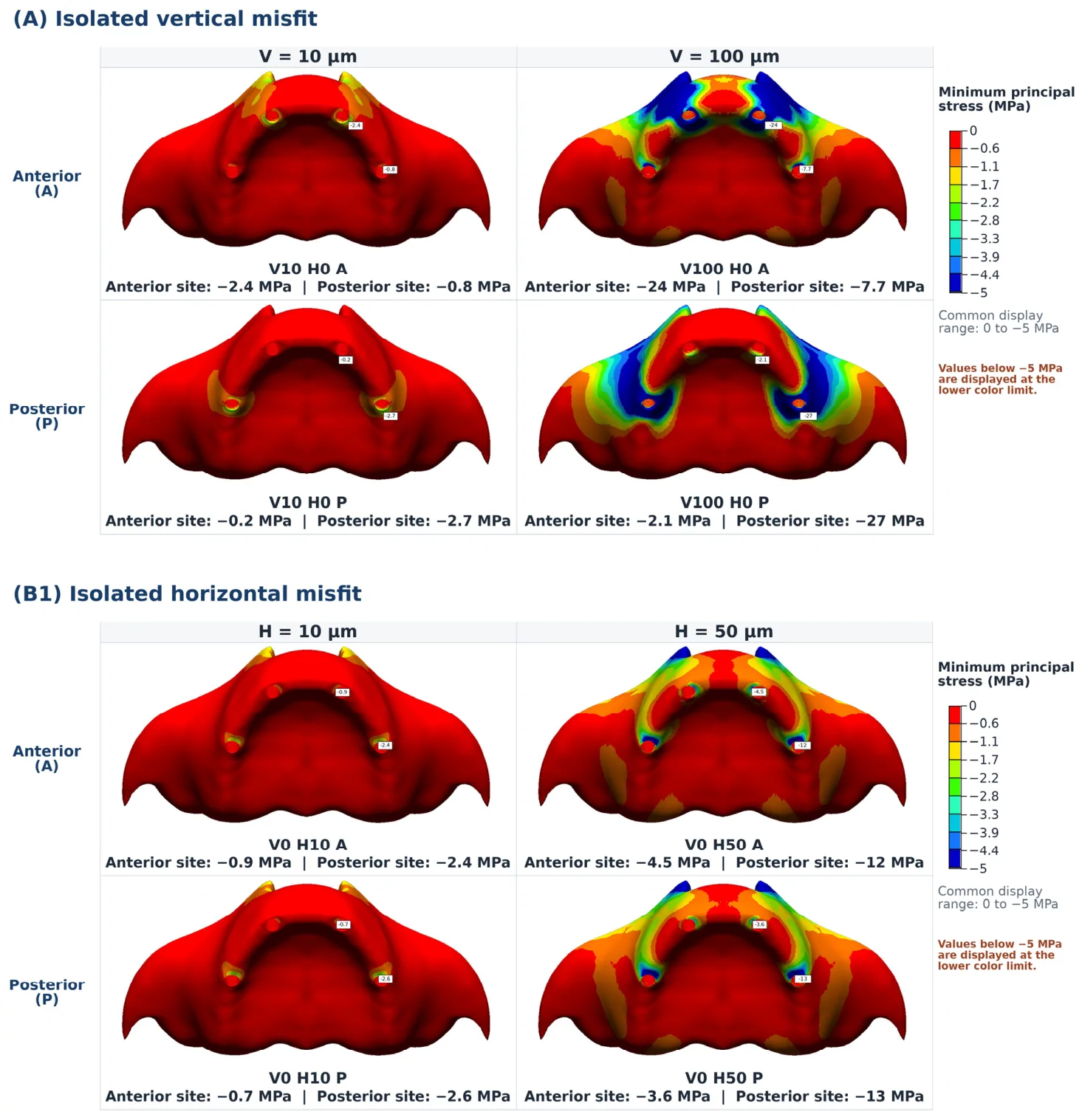

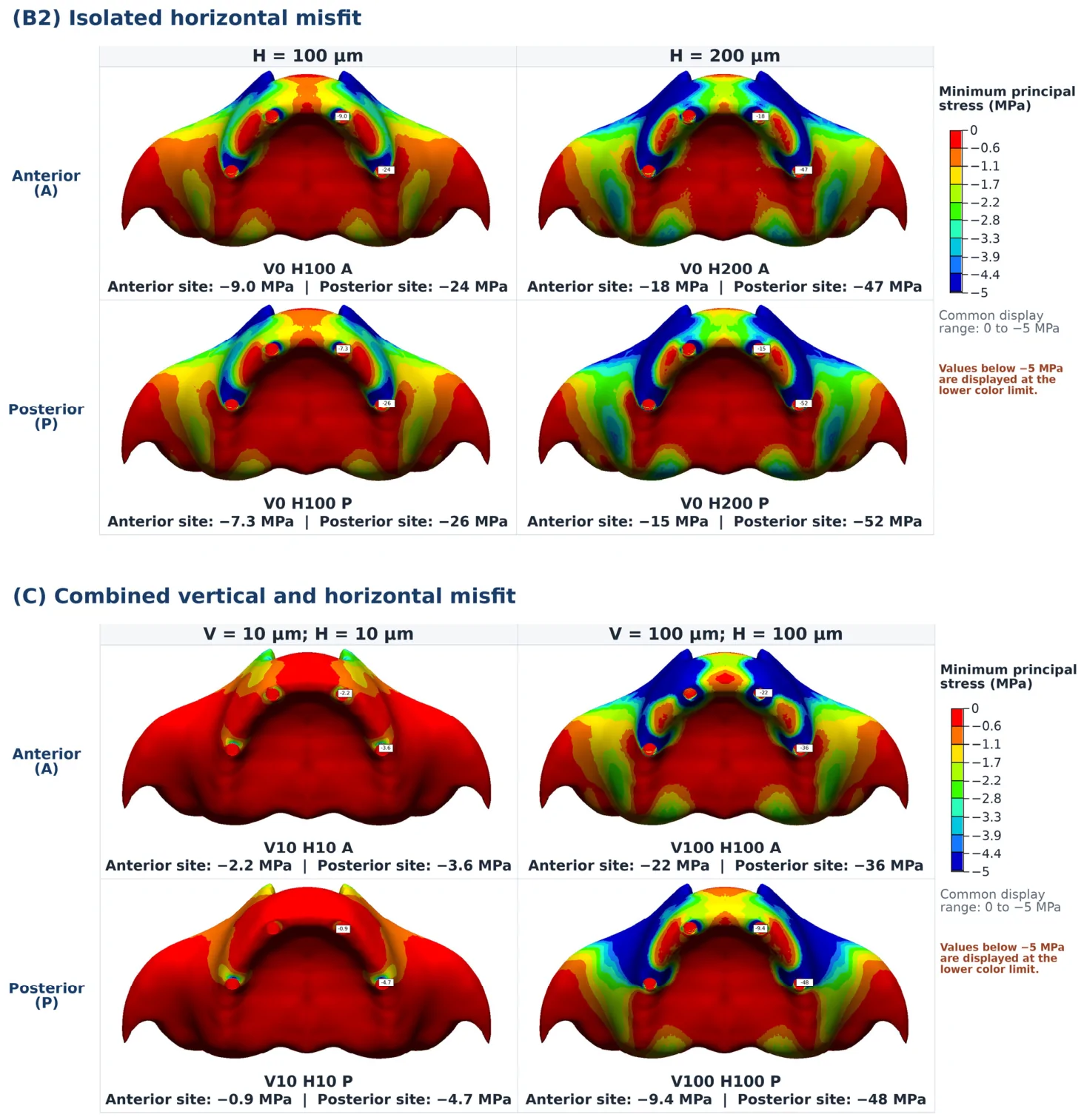
Minimum principal stress distributions in the peri-implant bone for (**A**) isolated vertical, (**B1**,**B2**) isolated horizontal, and (**C**) combined misfit scenarios. Stress values are expressed in MPa. Boxed numbers indicate the site-specific stress values.

**Figure 7:**
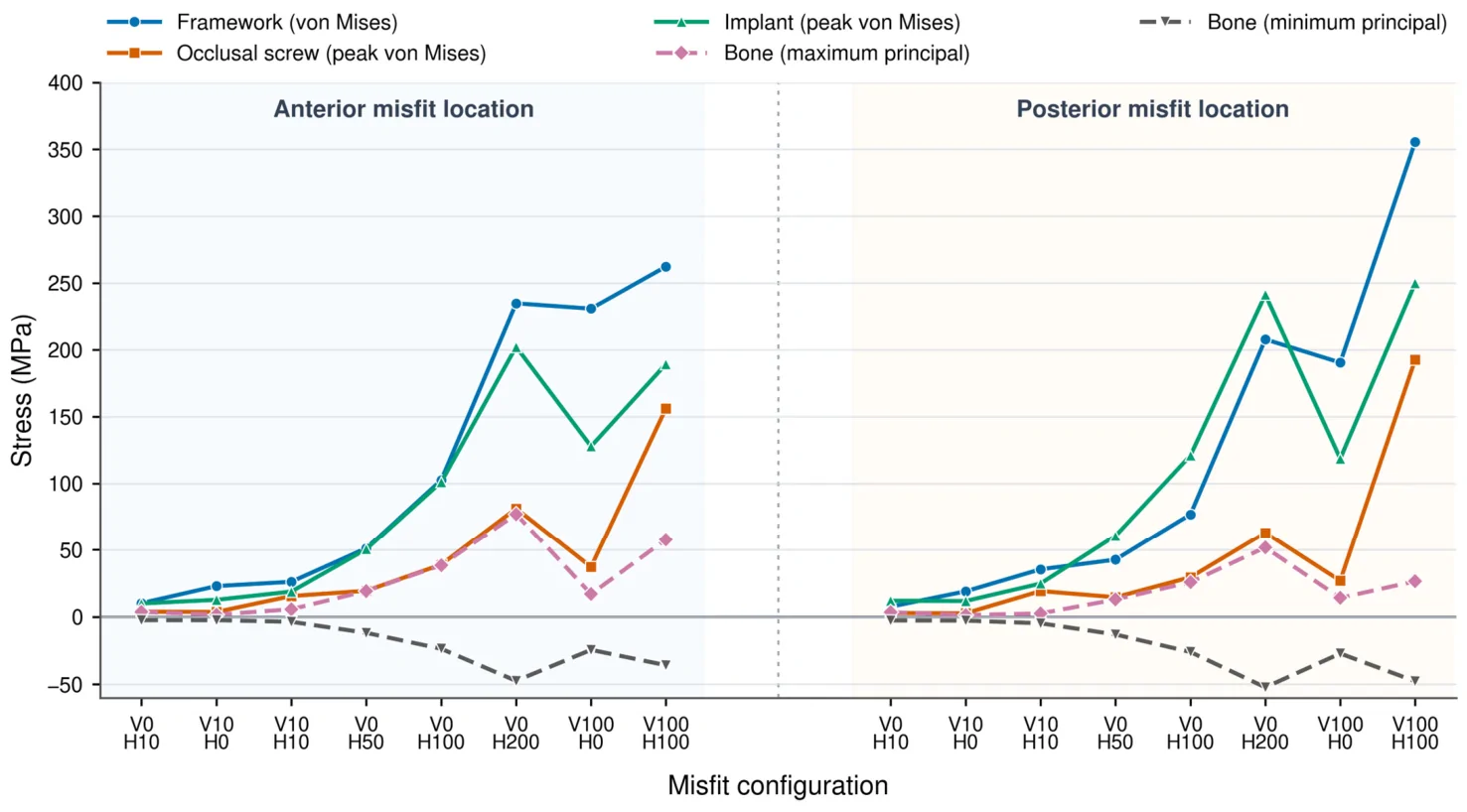
Peak stresses across anterior and posterior misfit scenarios, showing von Mises stresses in the framework, occlusal screws, and implants and principal stresses in peri-implant bone (MPa).

In the peri-implant bone, both maximum principal stress and the magnitude of minimum principal stress increased with isolated horizontal misfit magnitude. Unlike the framework, screws, and implants, the bone-stress extrema occurred in the V0 H200 conditions: the highest tensile stress was 77 MPa at the posterior bone site in V0 H200 A, and the greatest compressive stress was −52 MPa at the posterior site in V0 H200 P. Thus, the greatest bone response did not always coincide with the misfit location (Figure 5 and Figure 6). Peak stress trends across all scenarios are summarized in Figure 7.

## 4. Discussion

This study evaluated how the types, magnitudes, and locations of misfits affect stress transfer in a full-arch screw-retained implant framework. Because stress patterns differed among scenarios, the null hypothesis was rejected. V0 H10, V10 H0, and V10 H10 represented reduced-magnitude misfits, rather than passive controls. V0 H0 was omitted because, without a geometric discrepancy, closure displacement, screw preload, or external loading, a passively fitting assembly would generate no misfit-induced stress [7].

At matched locations, vertical misfits produced greater framework stress than horizontal misfits at both 10 and 100 µm; for example, at the anterior site, the values were 231 and 103 MPa, respectively, under a 100 µm misfit. Holding magnitude and location constant allowed for isolation of the effects of discrepancy orientation and seating direction, rather than assuming mechanical equivalence. Vertical and horizontal closures likely influence different combinations of bending, axial deformation, and shear in the connected framework, although it should be noted that these modes were not quantified separately. Thus, although equal-sized discrepancies in different directions should not be assumed to be biomechanically equivalent, the results do not define direction-specific clinical tolerances.

No universal MPa threshold exists for framework stress in full-arch prostheses; the von Mises stress should be considered relative to material strength and fatigue resistance. Previous FEA-based research has shown that increasing horizontal misfit raises stress in the bar, screw, implant, and cortical bone [19], while experimental and numerical studies have reported associations between misfit and veneer fracture or chipping [20,26]. In this study, the peak misfit-related stress was greater in the framework than in the screws, indicating greater framework demand under the modeled conditions.

The zirconia superstructure was stiffer than the titanium components in the model (200 vs. 110 GPa). Through FREEZE contact, it formed a bonded composite with the framework, reducing local compliance and potentially increasing reaction forces and the transmission of seating deformation across the arch. Its independent contribution, however, cannot be determined without material and interface sensitivity analyses.

Peak cortical bone tensile and compressive stresses (approximately 77 and 52 MPa, respectively) remained below commonly cited strength ranges [27,28], although compressive stress increased markedly under the 200 µm horizontal and 100–100 µm combined misfit configurations. As bone adaptation is better characterized using strain-based mechanobiology than principal stress alone, these values should be considered to indicate relative demand and localization, and not long-term adaptation or clinical risk [29,30]. Volumetric framework misfit has been quantified in vitro [31], and studies [32,33] have examined peri-implant responses under static loading and ill-fitting prostheses; however, the long-term association with bone-related complications remains uncertain [34]. Our findings complement those of Abdelrehim et al. [5]; in particular, while their reported ranges indicate the absence of observed complications, the present idealized displacement-controlled FEA compared relative stress responses and did not establish failure thresholds.

Previous studies have generally evaluated vertical and horizontal misfit separately [19,35,36,37]. However, combined closure requires the correction of a vertical gap and horizontal offset within the same constrained assembly, combining vertical and lateral bending with shear. This multiaxial response likely contributes to the higher von Mises stresses. Although combined conditions also had greater resultant displacement than the corresponding isolated conditions, the results do not establish nonlinear synergy. Nevertheless, V100 H100 produced greater framework stress than V0 H200 despite its smaller resultant displacement, showing that the direction and constraint pattern cannot be neglected. Experimental evidence indicates that there is a direct relationship between vertical misfit and peri-implant strain during framework fitting [38]. However, the influence of screw tightening on the effect of a combined misfit remains unknown, as sequential tightening and nonlinear contact modeling were not performed. Together with the veneer cracking and torque loss effects reported under misfit [26,39,40], this study’s findings support the need to minimize combined discrepancies rather than judging acceptability from the visible gap magnitude alone.

Horizontal misfit has been associated with increased screw and implant stress [19,37]. In this context, closure transverse to the screw–abutment axis likely generates an eccentric reaction and local bending and shear at the discrepant connection. Under the bonded-interface idealization in this study, this reaction followed the screw–framework–abutment load path and led to stress concentration in the corresponding screw. Tightening torque and implant–abutment contact can also influence screw stress and microgap formation [41]. As preload, frictional thread contact, sequential tightening, and contact or micromobility at the intentionally discrepant interface were not modeled in this study, the reported screw values represent displacement-induced demand rather than total post-tightening stress; clinical tightening could alter interface contact and lead to stress redistribution. As the lever arm increases bending moments, posterior implants often experience greater demand under distal cantilever loading [42,43]. Such a mechanism was not directly reproduced in this study, because no occlusal load was applied. Therefore, posterior dominance was not uniform; in particular, isolated misfits produced greater framework stress when applied anteriorly, whereas the high-magnitude combined condition produced the greatest framework, screw, and implant stresses in the posterior configuration. Screw stress was localized at the discrepant connection, but peak tensile bone stress occurred posteriorly with an anterior 200 µm horizontal misfit. Thus, localization reflects the closure pathway, seated connections, framework continuity, component stiffness, and constraints—not cantilever alone—and the misfit site does not necessarily coincide with the maximum-stress site.

As the geometry, mesh, materials, boundary conditions, and interfaces were held constant, the most defensible findings of this study are the internally consistent comparative trends. In particular, stress rose across the isolated horizontal misfit series; combined misfits produced the highest framework and screw stresses and the maximum implant stress among the tested scenarios; and high stresses were not consistently confined to posterior sites. However, the absolute stresses, vertical–horizontal differences, anterior–posterior rankings, and clinical implications remain sensitive to displacement-controlled seating; omission of preload, sequential tightening, and functional or cyclic loading; and idealized contact, material, and boundary assumptions. Displacement-controlled misfit modeling has been performed previously [19], and misfit-induced peri-implant bone stress has been evaluated in multi-implant FEA-based research [44]. The uniform 2 mm cortical shell was not intended to represent patient- or site-specific maxillary anatomy, and may have affected the magnitude and localization of the bone stress results [22,24]. Sinus pneumatization may preclude parallel posterior placement in atrophic maxillae, and distal tilt was not tested. Finally, the vertical series was limited to 10 and 100 µm and, thus, a full dose–response relationship cannot be defined. Nonlinear contact and preload analyses, sensitivity testing, and experimental or clinical validation will be required before the values reported here can be translated into thresholds or indicators of complication risk.

## 5. Conclusions

Within the limitations of this finite element model, combined misfit produced the highest framework and occlusal-screw stresses among the tested scenarios, whereas implant and peri-implant bone responses varied with misfit magnitude and location. These findings describe comparative biomechanical trends and stress localization patterns rather than clinical acceptability thresholds. Clinically, combined misfits warrant particular attention during framework fit assessment before definitive seating.

## Figures and Tables

**Table 1:** Misfit scenarios and configurations.

Group	Scenario Code and Misfit Location	Vertical Misfit, V (µm)	Horizontal Misfit, H (µm)	Misfit Configuration
V0 H10	V0 H10 A—anterior V0 H10 P—posterior	0	10	Isolated horizontal
V10 H0	V10 H0 A—anterior V10 H0 P—posterior	10	0	Isolated vertical
V10 H10	V10 H10 A—anterior V10 H10 P—posterior	10	10	Combined vertical and horizontal
V0 H50	V0 H50 A—anterior V0 H50 P—posterior	0	50	Isolated horizontal
V0 H100	V0 H100 A—anterior V0 H100 P—posterior	0	100	Isolated horizontal
V0 H200	V0 H200 A—anterior V0 H200 P—posterior	0	200	Isolated horizontal
V100 H0	V100 H0 A—anterior V100 H0 P—posterior	100	0	Isolated vertical
V100 H100	V100 H100 A—anterior V100 H100 P—posterior	100	100	Combined vertical and horizontal

**Table 2:** Nominal element sizes and mesh characteristics of the model components.

Component/Scenario	Surface-Element Size Range (mm)	Number of Nodes	Number of Tetrahedral Elements
Shared model components			
Cortical bone	0.10–0.25	37,506	138,064
Trabecular bone	0.10–0.25	67,680	274,723
Implant component set	0.10	36,667	146,151
Multi-unit abutment component set	0.10	16,862	66,574
Occlusal screw component set	0.10	3626	12,761
Zirconia prosthesis	0.10	52,952	218,828
Scenario-specific titanium framework meshes			
Titanium framework—V0 H10 A	0.10	67,377	280,373
Titanium framework—V0 H10 P	0.10	66,773	277,907
Titanium framework—V10 H0 A	0.10	66,492	276,336
Titanium framework—V10 H0 P	0.10	66,972	278,752
Titanium framework—V10 H10 A	0.10	67,369	280,418
Titanium framework—V10 H10 P	0.10	66,766	277,896
Titanium framework—V0 H50 A	0.10	66,695	277,059
Titanium framework—V0 H50 P	0.10	66,893	278,141
Titanium framework—V0 H100 A	0.10	65,842	272,917
Titanium framework—V0 H100 P	0.10	67,043	278,434
Titanium framework—V0 H200 A	0.10	66,232	274,452
Titanium framework—V0 H200 P	0.10	66,870	277,191
Titanium framework—V100 H0 A	0.10	66,417	276,741
Titanium framework—V100 H0 P	0.10	66,905	278,654
Titanium framework—V100 H100 A	0.10	65,942	273,821
Titanium framework—V100 H100 P	0.10	66,559	277,287

Note: Counts for the implant, multi-unit abutment, and occlusal screw represent one symmetric component set; two identical sets were included in the four-implant model. Framework counts varied slightly because each discrepancy geometry was meshed separately.

## Data Availability

The original contributions presented in the study are included in the article, further inquiries can be directed to the corresponding author.
